# EARLY IDENTIFICATION OF COGNITIVE IMPAIRMENT AMONG VULNERABLE OLDER ADULTS LIVING AT HOME

**DOI:** 10.1093/geroni/igz038.3009

**Published:** 2019-11-08

**Authors:** Maria T Brown, Sharon A Brangman

**Affiliations:** 1 Syracuse University, Syracuse, New York, United States; 2 SUNY Upstate Medical University, Syracuse, New York, United States

## Abstract

African Americans are twice as likely as Caucasians to develop Alzheimer’s disease and related dementias (AD/D), are at greater risk of frailty if they have AD/D, and are less likely to seek a diagnosis before experiencing a crisis. This collaborative community-based project aimed to evaluate the processes and outcomes of adding a Mini-Cog© screening to existing Area Agency on Aging (AAA) assessments performed by a neighborhood advisor in a lower-income African American neighborhood in a mid-sized northeastern city. AAA clients receive initial in-home screenings to assess their need for income-related services to help them continue living safely in the community, but are not screened for potential cognitive issues that may exacerbate or be masked by the very conditions that AAAs aim to address. This pilot was designed to introduce a mechanism for the early identification of previously undetected cognitive impairment, provide referrals for comprehensive cognition screening at a Center of Excellence for Alzheimer’s Disease (CEAD), and increase access to needed health and community support services. We hypothesized that adding the Mini-Cog© to existing AAA assessments would increase the early identification of vulnerable older African Americans with or at risk of AD/D. Process outcomes included challenges encountered when engaging community caregivers on the project team, learning to interpret Mini-Cog© results, the labor-intensive nature of scheduling Mini-Cog© assessments with existing clients, addressing client stigma and fear of AD/D, and client resistance to comprehensive testing. This presentation reviews process and outcome evaluation data from the pilot, and discusses implications for expanding the intervention.

